# Spatial distribution of SARS-CoV-2 infection in schools, South Korea

**DOI:** 10.1017/S095026882200173X

**Published:** 2021-11-08

**Authors:** Young Hwa Lee, Young June Choe, Hyunju Lee, Eun Hwa Choi, Young-Joon Park, Hyun Joo Jeong, Myoungyoun Jo, Heegwon Jeong

**Affiliations:** 1 Allergy Immunology Center, Korea University, Seoul, Korea; 2 Korea University Anam Hospital and Korea University College of Medicine, Seoul, Korea; 3 Seoul National University College of Medicine, Seoul, Korea; 4 Korea Disease Control and Prevention Agency, Cheongju, Korea; 5 Korea Educational Environment Protection Agency, Cheongju, Korea; 6 Korea Ministry of Education, Sejong, Korea

**Keywords:** COVID-19, risk factors, South Korea, spatial

## Abstract

Identification of geographical areas with high burden of severe acute respiratory syndrome coronavirus 2 (SARS-CoV-2) transmission in schools using spatial analyses has become an important tool to guide targeted interventions in educational setting. In this study, we aimed to explore the spatial distribution and determinants of coronavirus disease 2019 (COVID-19) among students aged 3–18 years in South Korea. We analysed the nationwide epidemiological data on laboratory-confirmed COVID-19 cases in schools and in the communities between January 2020 and October 2021 in South Korea. To explore the spatial distribution, the global Moran's I and Getis-Ord's G using incidence rates among the districts of aged 3–18 years and 30–59 years. Spatial regression analysis was performed to find sociodemographic predictors of the COVID-19 attack rate in schools and in the communities. The global spatial correlation estimated by Moran's I was 0.647 for the community population and 0.350 for the student population, suggesting that the students were spatially less correlated than the community-level outbreak of SARS-CoV-2. In schools, attack rate of adults aged 30–59 years in the community was associated with increased risk of transmission (*P* < 0.0001). Number of students per class (in kindergartens, primary schools, middle schools and high schools) did not show significant association with the school transmission of SARS-CoV-2. In South Korea, COVID-19 in students had spatial variations across the country. Statistically significant high hotspots of SARS-CoV-2 transmission among students were found in the capital area, with dense population level and high COVID-19 burden among adults aged 30–59 years. Our finding suggests that controlling community-level burden of COVID-19 can help in preventing SARS-CoV-2 infection in school-aged children.

## Introduction

As the waves of coronavirus disease 2019 (COVID-19) pandemic continue, prevention of severe acute respiratory syndrome coronavirus 2 (SARS-CoV-2) transmission in students require special attention. COVID-19 has caused a profound disruption of care and education in children, affecting significant burden on their growth and development [1]. According to a global estimate, COVID-19-related early childhood care and education disruptions resulted in 10 million children falling off track in their development [2].

Schools seemed to play minor role in SARS-CoV-2 transmission among the students. In the U.S., fewer than 1% of students were identified as having school-related COVID-19 [3]. In South Korea, less than 8% of the school-aged students with COVID-19 were infected from schools [4]. These findings can be attributed to community-related factors, and school-based COVID-19 response measures [5]. These factors have also favoured the occurrence of paediatric COVID-19 cases in clusters or ‘hotspots’ across geographic settings [6]. Thus, identification of geographical areas with high burden of SARS-CoV-2 transmission in schools has become an important tool to guide targeted interventions in educational settings. However, no studies have yet focused on social determinants associated with COVID-19 in school settings.

Following the delayed opening of schools in early 2020 in South Korea, a step-wise opening of schools was made from May to June 2020 in conjunction with the national COVID-19 pandemic response plan [7]. Between June and October of 2021, different on-and-off schooling policy was introduced in different regions, because of the difference in local incidence of COVID-19 [4].

In this study, we investigate the spatial distribution of COVID-19 among students aged 3–18 years, and explore the association of social determinants affecting SARS-CoV-2 transmission in South Korea.

## Methods

### Data sources

South Korea covers 100 431 km^2^ and has a population of approximately 51 million (51 333 253) in 2021 [8]. It consists of 17 provinces (si-do) divided into 250 districts (si-gun-gu) (Supplementary Fig. S1). The student population is comprised of children in early childhood education, primary and secondary education in Korea and is estimated at 7.2 million (7 212 784) in 2021, around 14% of the total population.

This was a retrospective, observational study comprised of all school-attending children at schools (K-12) who were diagnosed with COVID-19 through laboratory testing (reverse transcription polymerase chain reaction) in Korea from 19 January 2020 to 26 October 2021. Individual school's surveillance set linked to the Central Disease Control Headquarters' database was used to identify the epidemiologic trend of COVID-19 in schools and community in Korea. The linked database was derived from epidemiologic investigation files, collected through a legally mandated public health investigation under the authority of the Korean Infectious Diseases Control and Prevention Act (No. 12444 and No. 13392). Confirmed COVID-19 cases were identified from individual-level case reports submitted to the schools and to the health authorities. The incidence density on new cases from corresponding age group during each epidemiologic week was calculated. Following the merging of datasets, personal identifiable data were deleted and were not included in the analyses. Given the >99% school attendance rate in Korea, we defined (A) community infection as, those aged 0–2 years and >18 years with COVID-19; and (B) student infection as, those aged 3–18 years with COVID-19. We calculated the SARS-CoV-2 attack rates by age in each district using mid-year population data from the Korean National Statistics Office. Sociodemographic data on population density, class size of each education levels of early childhood, primary, secondary, number of private tutoring institutes per 1000 persons, percentage of foreign citizens, COVID-19 vaccine coverage rates for each district were retrieved.

We calculated the SARS-CoV-2 attack rates by age in each district using mid-year population data from the Korean National Statistics Office. Sociodemographic data on population density, class size of each education levels of early childhood, primary, secondary, number of private tutoring institutes per 1000 persons, percentage of foreign citizens, COVID-19 vaccine coverage rates for each district were retrieved.

### Statistical analysis

An epidemic curve of weekly COVID-19 confirmed cases from the 4^th^ week in 2020 to the 44^th^ week in 2021 and an epidemic curve from the student population during the period from the 21^st^ week in 2020 (after in-person schooling was initiated) to the 36^th^ week in 2021 were drawn to reveal peaks, and the percentage of student cases over total cases was plotted to identify the trend.

To examine the spatial distribution of incidence rates among the districts and their spatial autocorrelation, we visualised the district incidence rates using a 10-color scale and calculated the global Moran's index. To find local indicators of spatial association, the local Moran's index and Getis-Ord's index, which display ‘hot spots’ (high values next to high, HH) and ‘cold spots’ (low values next to low, LL) clustering, were calculated.

Spatial regression analysis was performed to find sociodemographic predictors of the COVID-19 attack rate at the district level. The spatial lag and spatial error model are an extension of the traditional ordinary least square regression model that includes the spatial dependency of variables or errors in the model. The spatial lag model takes the following form:
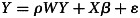
where values of the dependent variable in neighbouring locations (*WY*) are included as an extra explanatory variable. The spatial error model takes the following form:
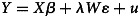
where values of the residuals in neighbouring locations 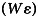 are included as an extra term in the equation.

We used different sociodemographic factors to predict the COVID-19 attack rates for the community and the students: population density, percentage of the aged 3–18 years old (students), percentage of the aged 30–59 years old (adults), percentage of foreign citizens and vaccine coverage for the community infections; and population density, attack rate of the aged 30–59 years old, number of students per class (kindergarten, primary-, middle- and high school each), number of private institute per 1000 persons, percentage of foreign citizens, and COVID-19 vaccine coverage in adult population (COVID-19 vaccination in children has not been started before October 2021) for the student infections.

We used GeoDa software (version 1.20, The University of Chicago, IL, USA) to visualise maps of incidence rates and local clusters and to conduct the spatial regression analyses. All other statistical analyses were performed using SAS software, version 9.3 (SAS Institute, Cary, NC, USA).

### Ethics statement

This study was reviewed and was approved by the Institutional Review Board of Korea University Anam Hospital (IRB No. 2021AN0314).

## Results

### Temporal trend

**Fig. 1.**
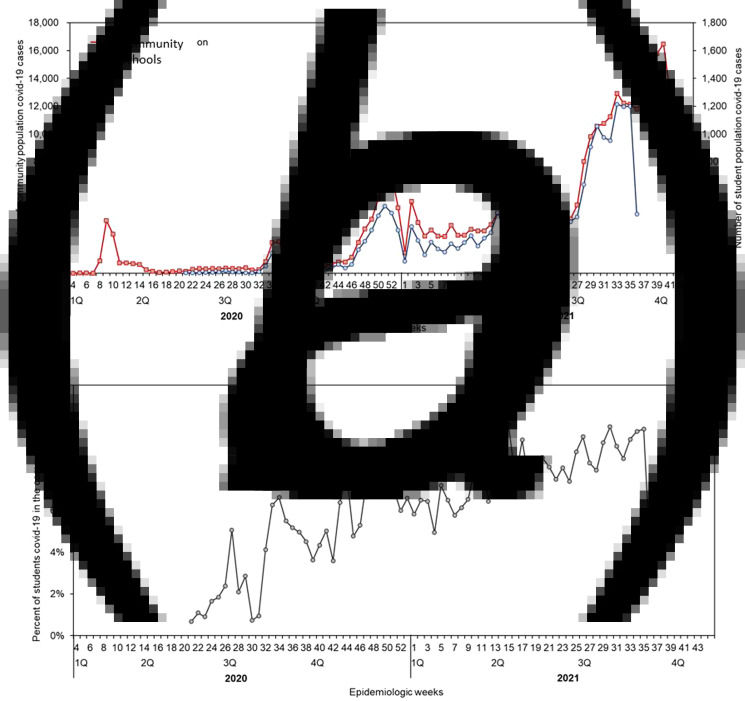
Epidemic curve of weeks COVID-19 cases, South Korea, January 2020 – October 2021. (a) is for community and schools' epidemic curve, and (b) is for per cent of students with COVID-19 among the community population. The incidences in the last week among the community and schools were truncated by Tuesday (26 Oct 2021 and 31 Aug 2021, respectively) so the last dip in the curve needs careful interpretation.

Figure 1(a) shows weekly COVID-19 incidences of 353 107 community infections and 19 215 student infections during the period from the 4^th^ week in 2020 to the 44^th^ week in 2021. The trends in incidence showed a similar pattern between both populations with four peaks in common at the 34–35th, 51–52nd week in 2020, and the 2nd, 33rd week in 2021 except for an additional peak from the students at the 30th week in 2021. The highest weekly incidence in the community was 16 461 on the 40th of 2021 and the one among the students was 1215 on the 33rd of 2021. The percentages of weekly COVID-19 incidence among the student infection to the community infection were shown in Figure 1(b).

### Spatial pattern

The COVID-19 incidence and attack rates in the community and the schools were summarised in Table 1. Seoul, the capital of South Korea, recorded the highest incidence of COVID-19 (*n* = 116 374) and attack rate (12.31 per 1000) in the community. The highest incidence of student COVID-19 incidences was in Gyeonggi (*n* = 6009), followed by Seoul (*n* = 4839) and Incheon (999); all in metropolitan area. Seoul had the highest attack rates in the schools (4.26 per 1000). The attack rate was highest in the community in secondary school-aged students (7.05 per 1000), followed by kindergarten children affected in community (5.32 per 1000) and primary school students affected in community (5.31 per 1000) (Supplementary Table S1).

**Table 1.** Attack rates of SARS-CoV-2 by geographic regions, South Korea, January 2020 – October 2021

Province (No. of districts)	School			Community		
	Population (n)	COVID-19 cases (n)	Attack rate (per 1000)	Population (n)	COVID-19 cases (n)	Attack rate (per 1000)
Total (250)	7 212 784	19 215	2.66	51 333 253	353 107	6.88
Seoul (25)	1 136 363	4839	4.26	9 453 878	116 374	12.31
Busan (16)	414 707	915	2.21	3 343 353	13 712	4.10
Daegu (8)	332 530	744	2.24	2 387 450	16 772	7.03
Incheon (10)	414 986	999	2.41	2 921 875	18 824	6.44
Gwangju (5)	228 897	347	1.52	1 438 868	5318	3.70
Daejeon (5)	214 365	665	3.10	1 449 628	7498	5.17
Ulsan (5)	173 983	525	3.02	1 124 041	5403	4.81
Gyeonggi (42)	2 047 572	6009	2.93	13 398 945	105 573	7.88
Sejong (1)	76 853	130	1.69	362 922	1350	3.72
Gangwon (18)	198 520	544	2.74	1 530 537	7106	4.64
Chungbuk (14)	224 290	412	1.84	1 591 246	7739	4.86
Chungnam (16)	309 978	589	1.90	2 109 102	10 332	4.90
Jeonbuk (15)	251 011	344	1.37	1 786 322	5043	2.82
Jeonnam (22)	244 780	245	1.00	1 833 021	3476	1.90
Gyeongbuk (24)	341 795	639	1.87	2 619 828	9530	3.64
Gyeongnam (22)	492 716	949	1.93	3 311 249	12 704	3.84
Jeju (2)	109 442	320	2.92	670 993	3082	4.59

**Fig. 2.**
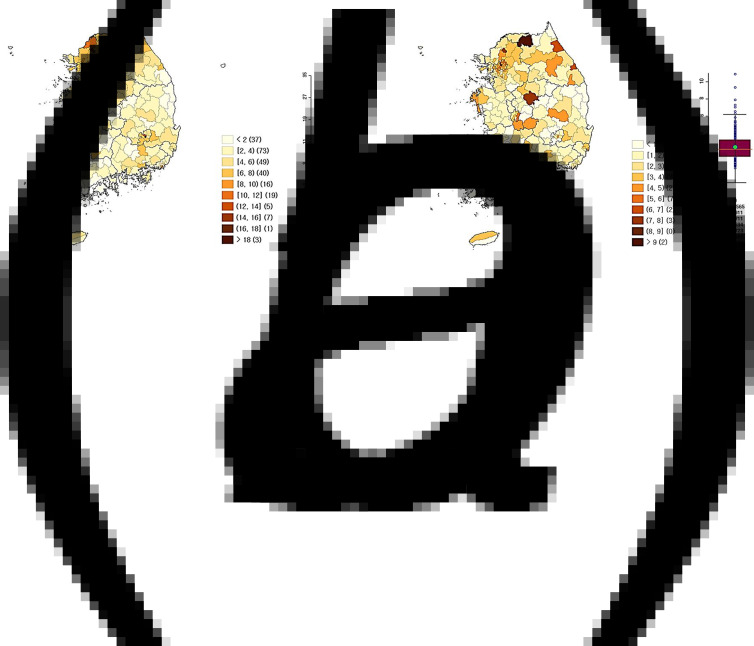
Attack rate per 1000 cases of COVID-19, South Korea, January 2020 – October 2021. (a) is for community and (b) is for schools.

Figure 2 shows the COVID-19 attack rates in the (A) community and (B) schools. Both in the community and in the schools, the highest attack rates were in the metropolitan area, located in the northwestern part of the country. The Supplementary Figure S2 shows the timely trend change in attack rates in the (A) community and (B) schools.

Table 2 shows global spatial autocorrelation analysis of SARS-CoV-2 attack rates by (A) school and (B) community. The global spatial correlation estimated by Moran's I was 0.647 for the community infection and 0.350 for the school infection, suggesting that the school was spatially less correlated than the community (Table 2).

**Table 2.** Global spatial autocorrelation analysis of SARS-CoV-2 attack rates by (A) school and (B) community, South Korea, January 2020 – October 2021

	School			Community		
	Moran's index	*Z*	*P*	Moran's index	*Z*	*P*
Total	0.350	8.568	0.001	0.647	15.946	0.001
1st quarter 2020	0.000	0.000	0.001	0.462	13.319	0.001
2nd quarter 2020	0.379	9.475	0.001	0.217	6.255	0.001
3rd quarter 2020	0.146	3.736	0.002	0.516	12.646	0.001
4th quarter 2020	0.150	4.047	0.001	0.392	9.703	0.001
1st quarter 2021	0.158	4.023	0.003	0.444	10.571	0.001
2nd quarter 2021	0.312	7.209	0.001	0.600	15.043	0.001
3rd quarter 2021	0.119	3.063	0.006	0.615	14.963	0.001
4th quarter 2021	0.000	0.000	0.001	0.517	12.619	0.001

**Fig. 3.**
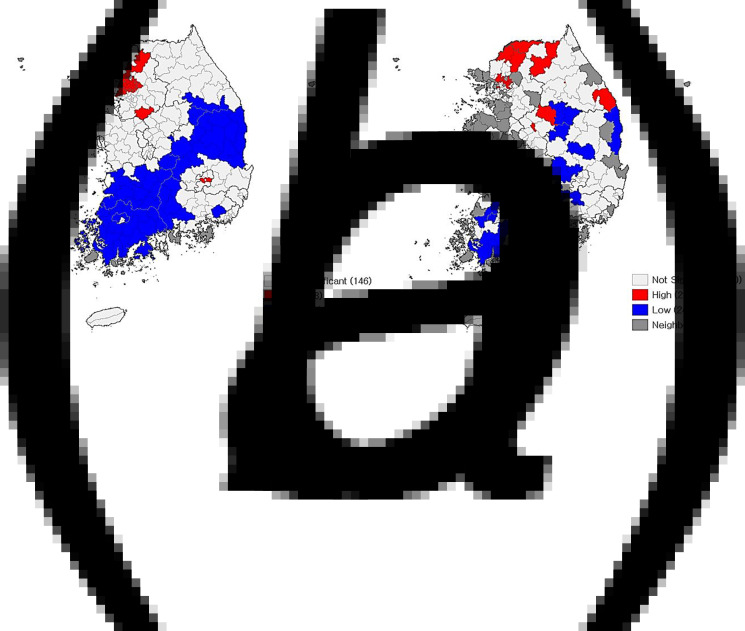
Hot Spot Analysis (Getis-Ord G*) results of COVID-19 attack rate per 1000 cases, South Korea, January 2020 – October 2021. (a) is for community and (b) is for schools.

Spatial clustering, demonstrating local autocorrelation, was examined by local Moran's index and Getis-Ord's G and showed similar patterns with hot spots and cold spots (Fig. 3). Among the community infection, hot spots were detected in Seoul and its neighbouring areas, and cold spots were detected in the southern belt. Among the school infections, hot spots and cold spots had a relatively scattered distributional pattern.

### Spatial regression analysis

Table 3 shows the COVID-19 attack rate in schools in relation to the sociodemographic predictors. Attack rate was highest in students of Seoul (4.26 per 1000), which had the highest attack rate of adults aged 30–59 years (13.61 per 1000), and population density (16 135.1 persons/km^2^). The number of students per class in early childhood, primary, lower secondary, upper secondary education ranged 3 to 18 between the regions (or districts) ranged between 17 and 28 between the regions; while the vaccine coverage rates also had small variations between the regions. Table 4 shows the COVID-19 attack rate in community in relation to the sociodemographic predictors. The highest percentage of persons aged 3–18 years in the total population was highest in Sejong (21.18%), followed by Jeju (16.31%) and Gyeongnam (14.88%). The community population attack rate in those three regions were at 7.88 per 1000 (Sejong), 4.59 per 1000 (Jeju) and 3.84 per 1000 (Gyeongnam), which was lower than that of Seoul (12.31 per 1000) and Incheon (6.44 per 1000).

**Table 3.** SARS-CoV-2 attack rate in schools and sociodemographic predictors by geographic regions, South Korea, January 2020 – October 2021

Province (No. of districts)	Attack rate of students (per 1000)	Attack rate of age 30–59 (per 1000)	Population density (no. of person/km^2^)	Class size (early childhood)	Class size (primary school)	Class size (middle school)	Class size (high school)	Number of private institute (per 1000)	Percentage of foreign citizens (%)	Adult 1-dose vaccine coverage rate (%)	Adult 2-dose vaccine coverage rate (%)
Total (250)	2.66	5.80	516.1	17.5	21.5	25.5	23.1	1.6	4.16	80.5	75.7
Seoul (25)	4.26	13.61	16 135.1	18.9	21.7	24.5	23.5	1.5	4.64	81.9	77.3
Busan (16)	2.21	4.77	4469.8	19.7	22.3	25.0	21.3	1.5	2.19	79.1	74.4
Daegu (8)	2.24	8.48	2786.3	20.2	22.4	24.2	22.9	1.7	2.11	76.9	72.0
Incheon (10)	2.41	5.87	2778.8	18.7	22.3	26.7	23.1	1.3	4.46	80.5	75.7
Gwangju (5)	1.52	3.93	2911.9	18.8	21.1	24.5	24.7	2.4	2.76	79.4	74.2
Daejeon (5)	3.10	4.94	2761.5	17.5	20.4	25.6	22.2	1.5	2.17	78.3	73.3
Ulsan (5)	3.02	4.83	1088.6	19.4	22.6	25.3	22.7	2.2	3.17	78.3	73.4
Gyeonggi (42)	1.69	8.41	675.7	15.5	20.9	22.7	22.6	2.2	5.31	73.3	68.8
Sejong (1)	2.93	3.87	1283.6	16.9	24.1	28.8	24.6	1.6	2.65	80.6	75.8
Gangwon (18)	2.74	4.56	91.4	14.9	17.0	22.9	21.3	1.4	2.41	81.1	76.4
Chungbuk (14)	1.84	5.30	215.9	15.1	19.5	23.6	23.4	1.4	4.48	82.2	77.1
Chungnam (16)	1.90	4.47	258.4	16.2	19.7	25.8	23.5	1.4	5.67	82.0	77.0
Jeonbuk (15)	1.37	2.52	227.6	14.2	18.0	23.2	22.0	2.2	3.40	81.7	77.0
Jeonnam (22)	1.00	1.87	152.6	13.6	17.4	22.3	20.2	1.5	3.74	83.1	78.7
Gyeongbuk (24)	1.87	3.34	140.6	17.1	19.9	21.5	20.9	1.4	3.73	80.0	75.0
Gyeongnam (22)	1.93	4.03	320.1	18.4	20.8	25.1	22.8	1.8	3.79	79.3	74.3
Jeju (2)	2.92	4.05	360.6	21.0	22.4	26.4	25.4	1.6	5.04	78.7	73.1

**Table 4.** SARS-CoV-2 attack rate in community and sociodemographic predictors by geographic regions, South Korea, January 2020 – October 2021

Province (No. of districts)	Attack rate in community population (per 1000)	Percentage of age 3–18 in population (%)	Percentage of age 30–59 in population (%)	Population density (no. of person/km^2^)	Percentage of foreign citizens (%)	Adult 1-dose vaccine coverage rate (%)	Adult 2-dose vaccine coverage rate (%)
Total (250)	6.88	14.05	45.68	516.1	4.16	80.5	75.7
Seoul (25)	12.31	12.02	46.63	16 135.1	4.64	81.9	77.3
Busan (16)	4.10	12.40	43.85	4469.8	2.19	79.1	74.4
Daegu (8)	7.03	13.93	45.31	2786.3	2.11	76.9	72.0
Incheon (10)	6.44	14.20	47.37	2778.8	4.46	80.5	75.7
Gwangju (5)	3.70	15.91	45.36	2911.9	2.76	79.4	74.2
Daejeon (5)	5.17	14.79	45.65	2761.5	2.17	78.3	73.3
Ulsan (5)	4.81	15.48	47.99	1088.6	3.17	78.3	73.4
Gyeonggi (42)	3.72	15.28	47.77	675.7	5.31	73.3	68.8
Sejong (1)	7.88	21.18	49.29	1283.6	2.65	80.6	75.8
Gangwon (18)	4.64	12.97	42.29	91.4	2.41	81.1	76.4
Chungbuk (14)	4.86	14.10	43.96	215.9	4.48	82.2	77.1
Chungnam (16)	4.90	14.70	43.84	258.4	5.67	82.0	77.0
Jeonbuk (15)	2.82	14.05	41.63	227.6	3.40	81.7	77.0
Jeonnam (22)	1.90	13.35	40.71	152.6	3.74	83.1	78.7
Gyeongbuk (24)	3.64	13.05	42.56	140.6	3.73	80.0	75.0
Gyeongnam (22)	3.84	14.88	45.09	320.1	3.79	79.3	74.3
Jeju (2)	4.59	16.31	45.42	360.6	5.04	78.7	73.1

Table 5 shows the spatial regression of sociodemographic predictors of COVID-19 attack rate in schools and community. In schools, attack rate of adults aged 30–59 years in the community was associated with increased risk of transmission (*P* < 0.0001). Number of students per class (in kindergartens, primary schools, middle schools and high schools) did not show significant association with the school transmission of SARS-CoV-2. In community, population density(*P* = 0.0007) and percentage of adults aged 30–59 in the community (*P* < 0.0001) were associated with increased risk of SARS-CoV-2 transmission; while percentage of children aged 3–18 years in the community was inversely associated with the risk (*P* < 0.0001). Vaccination coverage in adult population did not show association in both school and community transmission of SARS-CoV-2.

**Table 5.** Spatial regression of sociodemographic predictors of SARS-CoV-2 attack rates in schools, South Korea, January 2020 – October 2021

		Student populations				
Model	Variable	Coeff.	SE	*P*	Akiake information criterion	*R*^2^
Schools	Constant	12.5544	6.5620	0.0569	817.7580	0.4754
	Population density	0.0000	0.0000	0.0130		
	Attack rate of age 30–59	0.2493	0.0243	0.0000		
	Class size (early childhood)	−0.0926	0.0444	0.0380		
	Class size (primary school)	−0.0450	0.0521	0.3884		
	Class size (middle school)	0.1057	0.0431	0.0150		
	Class size (high school)	0.0003	0.0337	0.9933		
	No. of private institute per 1000 persons	−0.3175	0.1841	0.0859		
	Percentage of foreign citizens	−0.0859	0.0348	0.0143		
	1-dose vaccine coverage	−0.2444	0.4682	0.6021		
	2-dose vaccine coverage	0.1056	0.4344	0.8081		
	3-dose vaccine coverage	3.0962	1.2728	0.0157		
Community	Constant	−15.6523	14.1728	0.2705	1224.0200	0.5583
	Population density	0.0002	0.0000	0.0007		
	Percentage of age 3–18 in population	−57.8203	11.2856	0.0000		
	Percentage of age 30–59 in population	55.0022	7.3242	0.0000		
	Percentage of foreign citizens	0.3733	0.0732	0.0000		
	1-dose vaccine coverage	−0.2772	1.0552	0.7930		
	2-dose vaccine coverage	0.3334	0.9875	0.7359		
	3-dose vaccine coverage	−0.1973	2.7923	0.9437		

## Discussion

In this study, the distribution of SARS-CoV-2 transmission among students varied in South Korea. The Moran's *I* value 0.350 (*P* = 0.001) in schools indicated that there was a significant less clustering of COVID-19 in schools compared to community (Moran's *I*, 0.648, *P* = 0.001). The spatial analysis identified multiple clusters with the high-risk regions for COVID-19 in schools were the capital area of Seoul and neighbouring regions. These areas are densely populated settings, implying a high burden of SARS-CoV-2 transmission among adult population, which is in line with the previous reports [9, 10]. In the early phase of COVID-19 pandemic, young adults were found to be the primary source of transmission in the community, which partly explains the correlation between the population density and the COVID-19 in school-aged children [11]. However, our finding points different direction from other studies. In a modelling study using data on country-specific policies on NPIs from the Oxford COVID-19 Government Response Tracker, a decreasing trend over time in the R ratio was found following the introduction of school closure was shown [12]. Another modelling study that links NPI implementation dates to national case and death counts across 41 countries showed that closing schools was more effective than stay-at-home orders in slowing transmission of COVID-19 [13]. The reason for the difference is not clear, however, there are possibility that school-aged children may still gather around outside of the schools even if the schools are closed. The difference in level of activities and contact rates with the adults may have affected the overall attack rates in different age group among school-aged children [14, 15]. In spite of these conflicting results, modelling studies and observational studies are prone to bias. According to the best available evidence, including a number of quasi-experimental studies and our finding, schools are unlikely to have played a major role in the outbreak; however, school transmission may reflect the intensity of community transmission [16]. Our finding implicates that the control of COVID-19 epidemic in adult population can be more crucial in preventing SARS-CoV-2 infection in school-aged children.

This study showed that the COVID-19 incidence of adults age-group of 30–59 years, presumably the parents of school-aged children, were positively associated with increase in SARS-CoV-2 infection in the schools. The finding is in line with a systematic review where the risk of SARS-CoV-2 infection is significantly higher in children whose parents were infected [17]. The possible justification could be related to childcare in which parents play a critical role in childhood COVID-19 [18]. The other explanation could be that parents were more likely to be exposed to novel pathogen or new variants, thereby transmitting the infection to their children may be increased [19].

This study has several limitations. First, the observed clusters may have been underestimated because the data were derived from administrative data on place of residence, and do not contain true place where the transmission might have occurred. Second, the adult vaccination factors related to COVID-19 incidence have not been well demonstrated because of unavailability of adult vaccination coverage data at individual level. Lastly, the observed period was during when South Korea has introduced a strong social distancing policy, therefore children were likely to have limited social interaction with others. Given more of the community-spread of SARS-CoV-2 omicron variant since March 2022, there could be cases of school-driven community spread of COVID-19 outbreaks. Despite the limitations, our data have certain strengths. This is the first attempt to conduct exploratory data analysis on transmission of COVID-19 in school-aged children according to time and space from a country with a well-established COVID-19 surveillance system. Second, another strength of this study was its representativeness at national and regional levels, so it can be generalised to other places when investigating potential role of societal factors on school-children's vulnerability to COVID-19. The findings of this study may provide valuable policy implications for school health programme including COVID-19 mitigation.

In South Korea, COVID-19 incidence in schools showed spatial variations across the country. Statistically significant high hotspots of SARS-CoV-2 transmission among students were found in the capital area, with dense population level and high COVID-19 burden among adults aged 30–59 years. Our finding suggests that controlling community-level burden of COVID-19 can help in preventing SARS-CoV-2 infection in schools.

## Data Availability

Data are available by request through the Korea Disease Control and Prevention Agency. However, given the nature of this research and the fact that participants of this study did not agree for their interview data to be shared publicly, the data are not available.

## Supplementary material

For supplementary material accompanying this paper visit https://doi.org/10.1017/S095026882200173X. click here to view supplementary material
