# Supplementary material for: Spatial distribution of SARS-CoV-2 infection in schools, South Korea
Source: Epidemiol Infect. 2021 Nov 8;150:e194. doi: 10.1017/S095026882200173X (PMC9744459; doi:10.1017/S095026882200173X)
Supplement: Supplementary file 1 [file hygsup.zip › S095026882200173Xsup003.docx]

Supplementary Table 1. Attack rates of SARS-CoV-2 by age groups, South Korea, January 2020 –October 2021

| Province  (No. of districts) | School | | | | | | Community | | | | | |
| --- | --- | --- | --- | --- | --- | --- | --- | --- | --- | --- | --- | --- |
|  | Kindergartens,  3-6yrs | | Primary schools,  7-12yrs | | Secondary schools,  13-18yrs | | Kindergartens,  3-6yrs | | Primary schools,  7-12yrs | | Secondary schools,  13-18yrs | |
|  | COVID-19 cases (n) | Attack rate (per 1000) | COVID-19 cases (n) | Attack rate (per 1000) | COVID-19 cases (n) | Attack rate (per 1000) | COVID-19 cases (n) | Attack rate (per 1000) | COVID-19 cases (n) | Attack rate (per 1000) | COVID-19 cases (n) | Attack rate (per 1000) |
| Total (250) | 1328 | 0.83 | 7839 | 2.82 | 9882 | 3.50 | 8,548 | 5.32 | 14780 | 5.31 | 19919 | 7.05 |
| Seoul (25) | 242 | 0.98 | 1870 | 4.37 | 2673 | 5.81 | 2275 | 9.17 | 4121 | 9.63 | 5673 | 12.33 |
| Busan (16) | 63 | 0.66 | 395 | 2.47 | 446 | 2.80 | 290 | 3.04 | 604 | 3.77 | 693 | 4.36 |
| Daegu (8) | 57 | 0.78 | 273 | 2.17 | 411 | 3.07 | 240 | 3.29 | 510 | 4.05 | 877 | 6.56 |
| Incheon (10) | 67 | 0.72 | 448 | 2.79 | 478 | 2.95 | 546 | 5.90 | 916 | 5.71 | 1120 | 6.92 |
| Gwangju (5) | 29 | 0.60 | 122 | 1.40 | 195 | 2.10 | 121 | 2.49 | 267 | 3.06 | 331 | 3.56 |
| Daejeon (5) | 62 | 1.34 | 277 | 3.41 | 321 | 3.70 | 235 | 5.07 | 424 | 5.22 | 712 | 8.20 |
| Ulsan (5) | 58 | 1.45 | 224 | 3.29 | 241 | 3.66 | 197 | 4.91 | 283 | 4.16 | 325 | 4.93 |
| Gyeonggi (42) | 394 | 0.84 | 2541 | 3.19 | 3018 | 3.86 | 3,099 | 6.61 | 4889 | 6.14 | 6183 | 7.90 |
| Sejong (1) | 7 | 0.35 | 92 | 2.91 | 31 | 1.22 | 60 | 3.01 | 121 | 3.83 | 88 | 3.47 |
| Gangwon (18) | 33 | 0.79 | 239 | 3.17 | 271 | 3.33 | 177 | 4.25 | 325 | 4.31 | 455 | 5.59 |
| Chungbuk (14) | 38 | 0.76 | 154 | 1.78 | 219 | 2.49 | 159 | 3.19 | 305 | 3.52 | 510 | 5.81 |
| Chungnam (16) | 64 | 0.92 | 221 | 1.82 | 301 | 2.52 | 285 | 4.11 | 497 | 4.10 | 707 | 5.92 |
| Jeonbuk (15) | 22 | 0.43 | 152 | 1.58 | 169 | 1.63 | 117 | 2.29 | 239 | 2.48 | 398 | 3.85 |
| Jeonnam (22) | 37 | 0.72 | 120 | 1.27 | 88 | 0.89 | 109 | 2.11 | 184 | 1.94 | 208 | 2.11 |
| Gyeongbuk (24) | 52 | 0.68 | 251 | 1.91 | 321 | 2.41 | 190 | 2.48 | 363 | 2.76 | 629 | 4.71 |
| Gyeongnam (22) | 92 | 0.85 | 399 | 2.06 | 452 | 2.37 | 378 | 3.48 | 563 | 2.91 | 695 | 3.65 |
| Jeju (2) | 11 | 0.45 | 61 | 1.41 | 247 | 5.93 | 70 | 2.87 | 169 | 3.89 | 315 | 7.56 |
