# Supplementary figures and images for: Spatial distribution of SARS-CoV-2 infection in schools, South Korea
Source: Epidemiol Infect. 2021 Nov 8;150:e194. doi: 10.1017/S095026882200173X (PMC9744459; doi:10.1017/S095026882200173X)

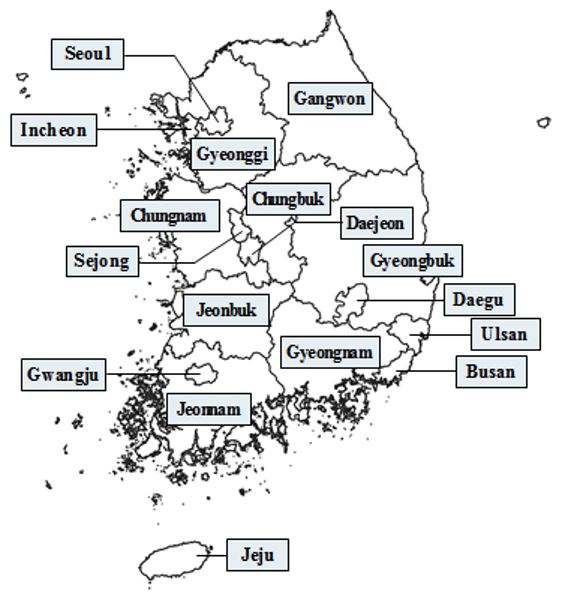

Supplement: Supplementary file 1 [file hygsup.zip › S095026882200173Xsup001.jpg]

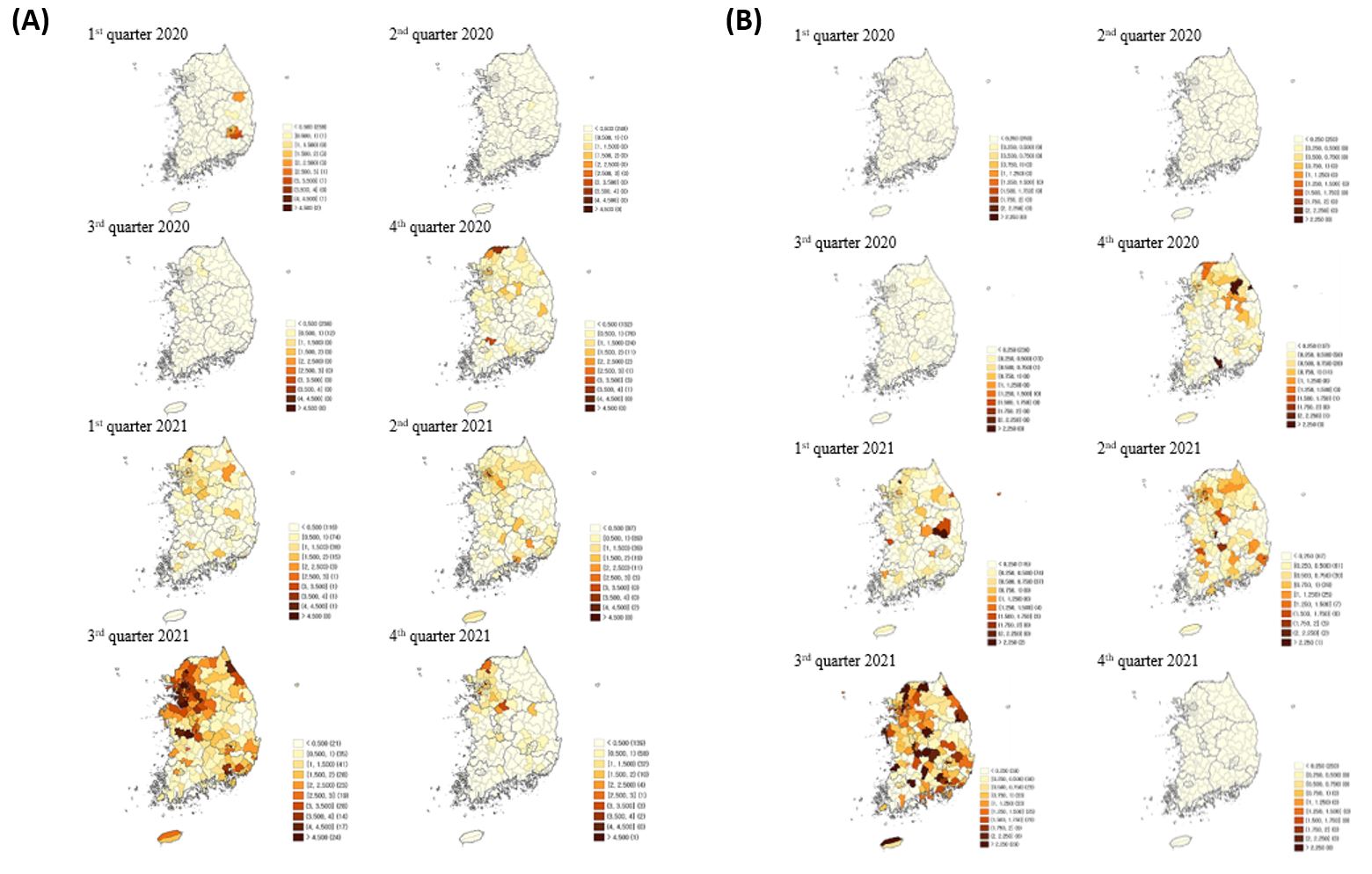

Supplement: Supplementary file 1 [file hygsup.zip › S095026882200173Xsup002.jpg]
